# Supplementary material for: Capturing cell heterogeneity in representations of cell populations for image-based profiling using contrastive learning
Source: PLoS Comput Biol. 2024 Nov 11;20(11):e1012547. doi: 10.1371/journal.pcbi.1012547 (PMC11611260; doi:10.1371/journal.pcbi.1012547)
Supplement: Attachment — Submitted filename: 142019_1_rebuttal_3100497_sh5j5q.pdf [file pcbi.1012547.s002.pdf]

Manuscript number: RC-2023-02268
Corresponding author(s): Shantanu Singh

General Statements

Thank you for your thorough and insightful reviews of our manuscript. We greatly appreciate the time and effort you have invested in providing such detailed and constructive feedback. Your comments have been very helpful towards improving the quality and clarity of our work.

We have carefully considered each of your suggestions and have made substantial revisions to the manuscript to address your concerns. Key changes include:

- Conducted an experiment isolating the effect of the contrastive formulation by applying CytoSummaryNet to averaged profiles, demonstrating its contribution to performance improvements.
- Performed an analysis progressively filtering out cells based on size and computing average profiles to confirm the interpretation that CytoSummaryNet prioritizes large, uncrowded cells.
- Improved the clarity of the Stain2-5 dataset descriptions and added a new section in the Experimental Setup to address this.
- Added Figure 2 to more clearly convey the data split strategy.
- Clarified the distinction between the replicate retrieval and mechanism of action prediction tasks, and updated the Methods section to explain the evaluation methodology for each task.
- Revised the terminology throughout the manuscript to more accurately describe our approach as self-supervised learning, reflecting its use of a pretext task (replicate retrieval) for learning an aggregation function.
- Improved the visual presentation of figures, including adding a graphical overview of the algorithm to Figure 1.

Our point-by-point responses detailing the changes made are included below. We believe these revisions have substantially strengthened the manuscript and effectively address the concerns raised during the review process.

Point-by-point description of the revisions

Reviewer 1

R1 Cell profiling is an emerging field with many applications in academia and industry. Finding better representations for heterogeneous cell populations is important and timely. However, unless convinced otherwise after a rebuttal/revision, the contribution of this paper, in our opinion, is mostly conceptual, but in its current form - not yet practical. This manuscript combined two concepts that were previously reported in the context of cell profiling, weakly supervised representations. Our expertise is in computational biology, and specifically applications of machine learning in microscopy.

In our revised manuscript, we have aimed to better clarify the practical contributions of our work by demonstrating the effectiveness of the proposed concepts on real-world datasets. We hope that these revisions and our detailed responses address your concerns and highlight the potential impact of our approach.

R1.1a. CytoSummaryNet is evaluated in comparison to aggregate-average profiling, although previous work has already reported representations that capture heterogeneity and self-supervision independently. To argue that both components of contrastive learning and sets representations are contributing to MoA prediction we believe that a separate evaluation for each component is required. Specifically, the authors can benchmark their previous work to directly evaluate a simpler population representation (PMID: 31064985, ref #13) - we are aware that the authors report a 20% improvement, but this was reported on a separate dataset. The authors can also compare to contrastive learning-based representations that rely on the aggregate (average) profile to assess and quantify the contribution of the sets representation.

We agree that evaluating the individual contributions of the contrastive learning framework and single-cell data usage is important for understanding CytoSummaryNet's performance gains.

To assess the impact of the contrastive formulation independently, we applied CytoSummaryNet to averaged profiles from the cpg0004 dataset. This isolated the effect of contrastive learning by eliminating single-cell heterogeneity. The experiment yielded a 32% relative improvement in mechanism of action retrieval, compared to the 68% gain achieved with single-cell data. These findings suggest that while the contrastive formulation contributes significantly to CytoSummaryNet's performance, leveraging single-cell information is crucial for maximizing its effectiveness. We have added a discussion of this experiment to the Results section:

"We conducted an experiment to determine whether the improvements in mechanism of action retrieval were due solely to CytoSummaryNet's contrastive formulation or also influenced by the incorporation of single-cell data. We applied the CytoSummaryNet framework to pre-processed average profiles from the 10 μM dose point data of Batch 1 (cpg0004 dataset). This approach isolated the effect of the contrastive architecture by eliminating single-cell data variability. We adjusted the experimental setup by reducing the learning rate by a factor of 100, acknowledging the reduced task complexity. All other parameters remained as described in earlier experiments.

This method yielded a less pronounced but still substantial improvement in mechanism of action retrieval, with an increase of 0.010 (32% enhancement - Table 1). However, this improvement was not as high as when the model processed single-cell level data (68% as noted above). These findings suggest that while CytoSummaryNet's contrastive formulation contributes to performance improvements, the integration of single-cell data plays a critical role in maximizing the efficacy of mechanism of action retrieval."

We don't believe comparing with PMID: 31064985 is useful: while the study showcased the usefulness of modeling heterogeneity using second-order statistics, its methodology is limited in scalability due to the computational burden of computing pairwise similarities for all perturbations, particularly in large datasets. Additionally, the study's reliance on similarity network fusion, while expedient, introduces complexity and inefficiency. We contend that this comparison does not align with our objective of testing the effectiveness of heterogeneity in isolation, as it primarily focuses on capturing second and first-order information. Thus, we do not consider this study a suitable baseline for comparison.

R1.1b. The evaluation metric of mAP improvement in percentage is misleading, because a tiny improvement for a MoA prediction can lead to huge improvement in percentage, while a much larger improvement in MoA prediction can lead to a small improvement in percentage. For example, in Fig. 4, MEK inhibitor mAP improvement of ~0.35 is measured as ~50% improvement, while a much smaller mAP improvement can have the same effect near the origins (i.e., very poor MoA prediction).

We agree that relying solely on percentage improvements can be misleading, especially when small absolute changes result in large percentage differences.

However, we would like to clarify two key points regarding our reporting of percentage improvements:

- We calculate the percentage improvement by first computing the average mAP across all compounds for both CytoSummaryNet and average profiling, and then comparing these averages. This approach is less susceptible to the influence of outlier improvements compared to calculating the average of individual compound percentage improvements.
- We report percentage improvements alongside their corresponding absolute improvements. For example, the mAP improvement for Stain4 (test set) is reported as 0.052 (60%).

To further clarify this point, we have updated the caption of Table 1 to explicitly state how the percentage improvements are calculated:

"*The improvements are calculated as* `mAP(CytoSummaryNet)-mAP(average profiling)`. *The percentage improvements are calculated as* `(mAP(CytoSummaryNet)-mAP(average profiling))/mAP(average profiling)`."

R1.1b. (Subjective) visual assessment of this figure does not show a convincing contribution of CytoSummaryNet representations of the average profiling on the test set (3.33 uM). This issue might also be relevant for the task of replicate retrieval. All in all, the mAP improvement reported in Table 1 and throughout the manuscript (including the Abstract), is not a proper evaluation metric for CytoSummaryNet contribution. We suggest reporting the following evaluations:

i. Visualizing the results of cpg0001 (Figs. 1-3) similarly to cpg0004 (Fig. 4), i.e., plotting the matched mAP for CytoSummaryNet vs. average profile.

ii. In Table 1, we suggest referring to the change in the number of predictable MoAs (MoAs that pass a mAP threshold) rather than the improvement in percentages. Another option is showing a graph of the predictability, with the X axis representing a threshold and Y-axis showing the number of MoAs passing it. For example see (PMID: 36344834, Fig. 2B) and (PMID: 37031208, Fig. 2A), both papers included contributions from the corresponding author of this manuscript.

Regarding the suggestion to visualize the results for compound group cpg0001 similarly to cpg0004, unfortunately, this is not feasible due to the differences in data splitting between the two datasets. In cpg0001, an MoA might have one compound in the training set and another in the test or validation set. Reporting a single value per MoA would require combining these splits, which could be misleading as it would conflate performance across different data subsets.

However, we appreciate the suggestion to represent the number of predictable MoAs that surpass a certain mAP threshold, as it provides another intuitive measure of performance. To address this, we have created a graph that visualizes the predictability of MoAs across various thresholds, similar to the examples provided in the referenced papers (PMID: 36344834, Figure 2B and PMID: 37031208, Figure 2A). This graph, with the x-axis depicting the threshold and the y-axis showing the number of MoAs meeting the criterion, has been added to Supplementary Material K.

R1.1c.i. "a subset of 18 compounds were designated as validation compounds" - 5 cross-validations of 18 compounds can make the evaluation complete. This can also enhance statistical power in figures 1-3.

We appreciate your suggestion and acknowledge the potential benefits of employing cross-validation, particularly in enhancing statistical power. While we understand the merit of cross-validation for evaluating model performance and generalization to unseen data, we believe the results as presented already highlight the generalization characterics of our methods.

Specifically, (the new) Figure 3 demonstrates the model's improvement over average profiling in both training and validation plates, supporting its ability to generalize to unseen compounds (but not to unseen plates).

While cross-validation could potentially enhance our analysis, retraining five new models solely for different validation set results may not substantially alter our conclusions, given the observed trends in Suppl Figure A1 and (the new) Figure 4, both of which show results across multiple stain sets (but a single train-test-validation split).

R1.1c.ii. Clarify if the MoA results for cpg0001 are drawn from compounds from both the training and the validation datasets. If so, describe how the results differ between the sets in text and graphs.

We confirm that the Mechanism of Action (MoA) retrieval results for cpg0001 are derived from all available compounds. It's important to note that the training and validation dataset split for the replicate retrieval task is different from the MoA prediction task. For replicate retrieval, we train using all available compounds and validate on a held-out set (see Figure 2). For MoA prediction, we train using the replicate retrieval task as the objective on all available compounds but validate using MoA retrieval, which is a distinct task. We have added a brief clarification in the main text to highlight the distinction between these tasks and how validation is performed for each:

“We next addressed a more challenging task: predicting the mechanism of action class for each compound at the individual well level, rather than simply matching replicates of the exact same compound (Figure 5). It's also important to note that mechanism of action matching is a downstream task on which CytoSummaryNet is not explicitly trained. Consequently, improvements observed on the training and validation plates are more meaningful in this context, unlike in the previous task where only improvements on the test plate were meaningful. For similar reasons, we calculate the mechanism of action retrieval performance on all available compounds, combining both the training and validation sets. This approach is acceptable because we calculate the score on so-called "sister compounds" only—that is, different compounds that have the same mechanism of action annotation. This ensures there is no overlap between the mechanism of action retrieval task and the training task, maintaining the integrity of our evaluation. ”

R1.1c.iii. "Mechanism of action retrieval is evaluated by quantifying a profile's ability to retrieve the profile of other compounds with the same annotated mechanism of action.". It was unclear to us if the evaluation of mAP for MoA identification can include finding replicates of the same compound. That is, whether finding a close replicate of the same compound would be included in the AP calculation. This would provide CytoSummaryNet with an inherent advantage as this is the task it is trained to do. We assume that this was

not the case (and thus should be more clearly articulated), but if it was - results need to be re-evaluated excluding same-compound replicates.

The evaluation excludes replicate wells of the same compound and only considers wells of other compounds with the same MoA. This methodology ensures that the model's performance on the MoA prediction task is not inflated by its ability to find replicates of the same compound, which is the objective of the replicate retrieval task. Please see the explanation we have added to the main text in our response to R1.1c.ii. Additionally, we have updated the Methods section to clearly describe this evaluation procedure:

“Mechanism of action retrieval is evaluated by quantifying a profile’s ability to retrieve the profile of ***different*** compounds with the same annotated mechanism of action.”

R1.2a. The description of Stain2-5 was not clear for us at first (and second) read. The information is there, but more details will greatly enhance the reader's ability to follow. One suggestion is explicitly stating that these "stains" partitioning was already defined in ref 26. Another suggestion is laying out explicitly a concrete example on the differences between two of these stains. We believe highlighting the differences between stains will strengthen the claim of the paper, emphasizing the difficulty of generalizing to the out-of-distribution stain.

We appreciate your feedback on the clarity of the Stain2-5 dataset descriptions; we certainly struggled to balance detail and concepts in describing these. We have made the following changes:

- Explicitly mentioned that the partitioning of the Stain experiments was defined in https://pubmed.ncbi.nlm.nih.gov/37344608/:
  “The partitioning of the Stain experiments have been defined and explained previously [21].”
- Moved an improved version of (now) Figure 2 from the Methods section to the main text to help visually explain how the stratification is done early on.
- Added a new section in the *Experimental Setup: Diversity of stain sets*, which includes a concrete example highlighting the differences between Stain2, and Stain5 to emphasize the diversity in experimental setups within the same dataset:

“Stain2-5 comprise a series of experiments which were conducted sequentially to optimize the experimental conditions for image-based cell profiling. These experiments gradually converged on the most optimal set of conditions; however, within each experiment, there were significant variations in the assay across plates. To illustrate the diversity in experimental setups within the dataset, we will highlight the differences between Stain2 and Stain5.

Stain2 encompasses a wide range of nine different experimental protocols, employing various imaging techniques such as Widefield and Confocal microscopy, as well as specialized conditions like multiplane imaging and specific stains like MitoTracker Orange. This subset also includes plates acquired with strong pixel binning instead of default imaging and plates with varying concentrations of dyes like Hoechst. As a result, Stain2 exhibits greater variance in the experimental conditions across different plates compared to Stain5.

In contrast, Stain5, the last experiment in the series, follows a more systematic approach, consistently using either confocal or default imaging across three well-defined conditions. Each condition in Stain5 utilizes a lower cell density of 1,000 cells per well compared to Stain2's 2,500 cells per well. Being the final experiment in the series, Stain5 had the least variance in experimental conditions.

For training the models, we typically select the data containing the most variance to capture the broadest range of experimental variation. Therefore, we chose Stain2-4 for training, as they represented the majority of the data and captured the most experimental variation. We reserved Stain5 for testing to evaluate the model's ability to generalize to new experimental conditions with less variance.

All StainX experiments were acquired in different passes, which may introduce additional batch effects.”

These changes aim to provide a clearer understanding of the dataset's complexity and the challenges associated with generalizing to out-of-distribution data.

R1.2b. What does each data point in Figures 1-3 represent? Is it the average mAP for the 18 validation compounds, using different seeds for model training? Why not visualize the data similarly to Fig. 4 so the improvement per compound can be clearly seen?

The data points in (the new) Figures 3,4,5 represent the average mAP for each plate, calculated by first computing the mAP for each compound and then averaging across compounds to obtain the average mAP per plate. We have updated the figure captions to clarify this:

"... (each data point is the average mAP of a plate) ..."

While visualizing the mAP per compound, similar to (the new) Figure 6 for cpg0004, could provide insights into compound-level improvements, it would require creating numerous additional figures or one complex figure to adequately represent all the stratifications we are analyzing (plate, compound, Stain subset). By averaging the data per plate across different stratifications, we aim to provide a clearer and more comprehensible overview of the trends and improvements while allowing us to draw conclusions about generalization.

Please note: this comment is related to the comment R1.1b (Subjective)

R1.2.c [On the topic of enhancing clarity and readability:] Justification and interpretation of the evaluation metrics.

Please refer to our response to comment R1.1b, where we have addressed your concerns regarding the justification and interpretation of the evaluation metrics.

R1.2d. Explicitly mentioning the number of MoAs for each datasets and statistics of number of compounds per MoA (e.g., average\median, min, max).

We have added the following to the *Experimental Setup: Data* section:

“A subset of the data was used for evaluating the mechanism of action retrieval task, focusing exclusively on compounds that belong to the same mechanism class. The Stain plates contained 47 unique mechanisms of action, with each compound replicated four times. Four mechanisms had only a single compound; the four mechanisms (and corresponding compounds) were excluded, resulting in 43 unique mechanisms used for evaluation. In the LINCS dataset, there were 1436 different mechanisms, but only 661 were used for evaluation because the remaining had only one compound.”

R1.2e. The data split in general is not easily understood. Figure 8 is somewhat helpful, however in our view, it can be improved to enhance understanding of the different splits. Specifically, the training and validation compounds need to be embedded and highlighted within the figure.

Thank you for highlighting this. We have completely revised the figure, now Figure 2 which we hope more clearly conveys the data split strategy.

Please note: this comment is related to the comment R1.2a.

R1.3a. Why was stain 5 used for the test, rather than the other stains?

Stain2-5 were part of a series of experiments aimed at optimizing the experimental conditions for image-based cell profiling using Cell Painting. These experiments were conducted sequentially, gradually converging on the most optimal set of conditions. However, within each experiment, there were significant variations in the assay across plates, with earlier iterations (Stain2-4) having more variance in the experimental conditions compared to Stain5. As Stain5 was the last experiment in the series and consisted of only three different conditions, it had the least variance. For training the models, we typically select the data containing the most variance to capture the broadest range of experimental variation. Therefore, Stain2-4 were chosen for training, while Stain5 was reserved for testing to evaluate the model's ability to generalize to new experimental conditions with less variance.

We have now clarified this in the *Experimental Setup: Diversity of stain sets* section. Please see our response to comment R1.2a. for the full citation.

R1.3b How were the 18 validation compounds selected?

20% of the compounds (n=18) were randomly selected and designated as validation compounds, with the remaining compounds assigned to the training set. We have now clarified this in the *Results* section:

“Additionally, 20% of the compounds (n=18) were randomly selected and designated as validation compounds, with the remaining compounds assigned to the training set (Supplementary Material H).”

R1.3c. For cpg0004, no justification for the specific doses selected (10uM - train, 3.33 uM - test) for the analysis in Figure 4. Why was the data split for the two dosages? For example, why not perform 5-fold cross validation on the compounds (e.g., of the highest dose)?

We chose to use the 10 µM dose point as the training set because we expected this higher dosage to consist of stronger profiles with more variance than lower dose points, making it more suitable for training a model. We decided to use a separate test set at a different dose (3.33 µM) to assess the model's ability to generalize to new dosages. While cross-validation on the highest dose could also be informative, our approach aimed to balance the evaluation of the model's generalization capability with its ability to capture biologically relevant patterns across different dosages.

This explanation has been added to the text:

“We chose the 10 µM dose point for training because we expected this high dosage to produce stronger profiles with more variance than lower dose points, making it more suitable for model training.”

“The multiple dose points in this dataset allowed us to create a separate hold-out test set using the 3.33 µM dose point data. This approach aimed to evaluate the model's performance on data with potentially weaker profiles and less variance, providing insights into its robustness and ability to capture biologically relevant patterns across dosages. While cross-validation on the 10 µM dose could also be informative, focusing on lower dose points offers a more challenging test of the model's capacity to generalize beyond its training conditions, although we do note that all compounds’ phenotypes would likely have been present in the 10 µM training dataset, given the compounds tested are the same in both.”

R1.3d. A more detailed explanation on the logic behind using a training stain to test MoA retrieval will help readers appreciate these results. In our first read of this manuscript we did not grasp that, we did in a second read, but spoon-feeding your readers will help.

This comment is related to the rationale behind training on one task and testing on another, which is addressed in our responses to comments R1.1.cii and R1.1.ciii.

R1.4 Assessment of interpretability is always tricky. But in this case, the authors can directly confirm their interpretation that the CytoSummaryNet representation prioritizes large uncrowded cells, by explicitly selecting these cells, and using their average profile representation to demonstrate that they achieve improved results. If this works, it could be applied as a general outlier removal strategy for cell profiling. "We identified the likely mechanism by which the learned CytoSummaryNet aggregates cells: the most salient cells are generally larger and more isolated from other cells, while the least salient cells appear to be smaller and more crowded, and tend to contain spots of high-intensity pixels (whether dying, debris or in some stage of cell division)." - doesn't such a mechanism should generalize to out-of-distribution data?

Thank you for the suggestion to directly confirm our interpretation by selecting large, uncrowded cells and using their average profile representation. We performed an analysis using five different plates from cpg0004 at the 10 µM dose point, resulting in approximately 50 wells to compute the replicate retrieval mAP.

We progressively filtered out cells based on a quantile threshold for Cells_AreaShape features (MeanRadius, MaximumRadius, MedianRadius, and Area), which were identified as important in our interpretability analysis, and then computed average profiles using the remaining cells before determining the replicate retrieval mAP. In the exclusion experiment, we gradually left out cells as the threshold increased, while in the inclusion experiment, we progressively included larger cells from left to right.

The results show that using only the largest cells does not significantly increase the performance. Instead, it is more important to include the large cells rather than only including small cells. The mAP saturates after a threshold of around 0.4, indicating that larger cells define the profile the most, and once enough cells are included to outweigh the smaller cell features, the profile does not change significantly by including even larger cells.

These findings support our interpretation that CytoSummaryNet prioritizes large, uncrowded cells. While this approach could potentially be used as a general outlier removal strategy for cell profiling, further investigation is needed to assess its robustness and generalizability across different datasets and experimental conditions.

We have created Supplementary Material L to report these findings and we additionally highlight them in the Results:

“To further validate CytoSummaryNet's prioritization of large, uncrowded cells, we progressively filtered cells based on Cells_AreaShape features and observed the impact on replicate retrieval mAP (Supplementary Material L). The results support our interpretation and highlight the key role of larger cells in profile strength.”

R1.5. Placing this work in context of other weakly supervised representations. Previous papers used weakly supervised labels of proteins / experimental perturbations (e.g., compounds) to improve image-derived representations, but were not discussed in this context. These include PMID: 35879608, https://www.biorxiv.org/content/10.1101/2022.08.12.503783v2 (from the same research groups and can also be benchmarked in this context), https://pubs.rsc.org/en/content/articlelanding/2023/dd/d3dd00060e , and https://www.biorxiv.org/content/10.1101/2023.02.24.529975v1. We believe that a discussion explicitly referencing these papers in this specific context is important.

While these studies provide valuable insights into improving cell population profiles using representation learning, our work focuses specifically on the question of single-cell aggregation methods. We chose to use classical features for our comparisons because they are the current standard in the field. This approach allows us to directly assess the performance of our method in the context of the most widely used feature extraction pipeline in practice. However, we see the value in incorporating them in future work and have mentioned them in the Discussion:

“Recent studies exploring image-derived representations using self-supervised and self-supervised learning [35][36] could inspire future research on using learned embeddings instead of classical features to enhance model-aggregated profiles.”

R1.minor1. "Because the improved results could stem from prioritizing certain features over others during aggregation, we investigated each cell's importance during CytoSummaryNet aggregation by calculating a relevance score for each" - what is the relevance score? Would be helpful to provide some intuition in the Results.

We have included more explanation of the relevance score in the Results section, following the explanation of sensitivity analysis (SA) and critical point analysis (CPA):

“SA evaluates the model's predictions by analyzing the partial derivatives in a localized context, while CPA identifies the input cells with the most significant contribution to the model's output. The relevance scores of SA and CPA are min-max normalized per well and then combined by addition. The combination of the two is again min-max normalized, resulting in the SA and CPA combined relevance score (see Methods for details).”

R1.minor2. Figure 1:
a. Colors of the two methods too similar
b. The dots are too close. It will be more easily interpreted if they were further apart.
c. What do the dots stand for?
d. We recommend considering moving this figure to the supp. material (the most important part of it is the results on the test set and it appears in Fig.2).

a. We chose a lighter and darker version of the same color as a theme to simplify visualization, as this theme is used throughout (the new) Figures 3,4,5.

b. We agree; we have now redrawn the figure to fix this.

c. Each data point is the average mAP of a plate. Please see our answer for R1.2b as well.

d. We believe that (the new) Figures 3,4,5 serve distinct purposes in testing various generalization hypotheses. We have added the following text to emphasize that the first figures are specifically about generalization hypothesis testing:

“We first investigated CytoSummaryNet’s capacity to generalize to out-of-distribution data: unseen compounds, unseen experimental protocols, and unseen batches. The results of these investigations are visualized in Figures 3, 4, and 5, respectively.”

R1.minor3 Figure 4: It is somewhat misleading to look at the training MoAs and validation MoAs embedded together in the same graph. We recommend showing only the test MoAs (train MoAs can move to SI).

We addressed this comment in R1.1c.ii. To reiterate briefly, there are no training, validation, or test MoAs because these are not used as labels during the training process. There is an option to split them based on training and validation compounds, which is addressed in R1.1c.ii.

R1.minor4 Figure 5

a. Why only Stain3? What happens if we look at Stains 2,3 and 4 together? Stain 5?

b. Should validation compounds and training compounds be analyzed separately?

c. Subfigure (d): it is expected that the data will be classified by compound labels as it is the training task, but for this to be persuasive I would like to see this separately on the training compounds first and then and more importantly on the validation compounds.

d. For subfigures (b) and (d): it appears there are not enough colors for d, which makes it partially not understandable. For example, the pink label in (d) shows a single compound which appears to represent two different MoAs. This is probably not the case, and it has two different compounds, but it cannot be inferred when they are represented by the same color.

e. For the Subfigure (e) - only 1 circle looks justified (in the top left). And for that one, is it not a case of an outlier plate that would perhaps need to be removed from analysis? Is it not good that such a plate will be identified?

a. We have addressed this point in the text, stating that the results are similar for Stain2 and Stain4. Stain5 represents an out-of-distribution subset because of a very different set of experimental conditions (see *Experimental Setup: Diversity of stain sets*). To improve clarity, we have revised the figure caption to reiterate this information:

"... Stain2 and Stain4 yielded similar results (data not shown). …"

b. For replicate retrieval, analyzing validation and training compounds separately is appropriate. However, this is not the case for MoA retrieval, as discussed in our responses to R1.1c.ii and R1.1c.i.

c. We have created the requested plot (below) but ultimately decided not to include it in the manuscript because we believe that (the new) Figures 3 and 4 are more effective for making quantitative comparative claims.

Top: training compounds (validation compounds grayed out); not all compounds are listed in the legend.
Bottom: validation compounds (training compounds grayed out).
Left: average profiling; Right: CytoSummaryNet

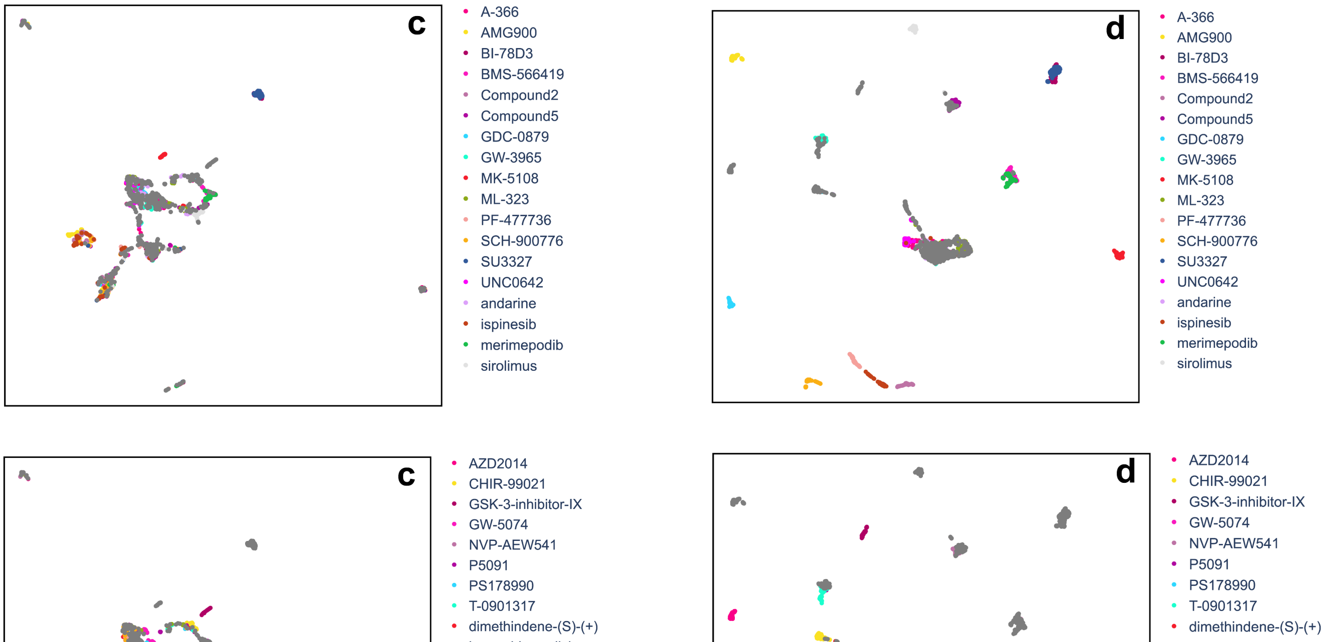

d. We agree with your observation and have addressed this issue by labeling the center mass as a single class (gray) and highlighting only the outstanding pairs in color. Please refer to the updated figure and our response to R3.6 for more details.

e. In the updated figure, we have revised the figure caption to focus solely on the annotation of same mechanism of action profile clusters, as indicated by the green ellipses. The annotation of isolated plate clusters has been removed (Figures 7e and 7f) to maintain consistency and avoid potential confusion. Despite being an outlier for Stain3, the plate (BR00115134bin1) clusters with Stain4 plates (Supplementary Figure F1, green annotated square inside the yellow annotated square), indicating it is not merely a noisy outlier and can provide insights into the out-of-sample performance of our model.

R1.minor5a. Discussion: "perhaps in part due to its correction of batch effects" - is this statement based on Fig. 5F - we are not convinced.

We appreciate the reviewer's scrutiny regarding our statement about batch effect correction. Upon reevaluation, we agree that this claim was not adequately substantiated by empirical data. We quantified the batch effects using comparison mean average precision for both average profiles and CytoSummaryNet profiles, and the statistical analysis revealed no significant difference between these profiles in terms of batch effect correction. Therefore, we have removed this theoretical argument from the manuscript entirely to ensure that all claims are strongly supported by the data presented.

R1.minor5b. "Overall, these results improve upon the ~20% gains we previously observed using covariance features" - this is not the same dataset so it is hard to reach conclusions - perhaps compare performance directly on the same data?

We have now explicitly clarified this is a different dataset. Please see our response to R1.1a for why a direct comparison was not performed. The following clarification can be found in the Discussion:

"These results improve upon the ~20% gains previously observed using covariance features [13] albeit on a different dataset, and importantly, CytoSummaryNet effectively overcomes the challenge of recomputation after training, making it easier to use."

Reviewer 2

R2.1 The authors present a well-developed and useful algorithm. The technical motivation and validation are very carefully and clearly explained, and their work is potentially useful to a varied audience.

That said, I think the authors could do a better job, especially in the figures, of putting the algorithm in context for an audience that is unfamiliar with the cell painting assay. (a) For example, a figure towards the beginning of the paper with example images might help to set the stage. (b) Similarly a schematic of the algorithm earlier in the paper would provide a graphical overview. (c) For the sake of a biologically inclined audience, I would consider labeling the images in the caption by cell type and label.

Thank you for your valuable suggestions on improving the accessibility of our figures for readers unfamiliar with the Cell Painting assay. We have made the following changes to address your comments:

a. and b. To provide visual context and a graphical overview of the algorithm, we have moved the original Figure 7 to Figure 1. This figure now includes example images that help readers new to the Cell Painting assay.

c. We have added relevant details to the example images in (the new) Figure 1

R2.2 The interpretability results were intriguing. The authors might consider further validating these interpretations by removing weakly informative cells from the dataset and retraining. Are the cells so uninformative that the algorithm does better without them, or are they just less informative than other cells?

Please see our responses to R1.4 and R3.0

R2.3 As far as I can tell, the authors only oblique state whether the code associated with the manuscript is openly available. Posting the code is needed for reproducibility. I would provide not only a github, but a doi linked to the code, or some other permanent link.

We have now added a Code Availability and Data Availability section, clearing stating that the code and data associated with the manuscript are openly available.

R2.4 Incorporating biological heterogeneity into machine-learning driven problems is a critical research question. Replacing means/modes and such with a machine learning framework, the authors have identified a problem with potentially wide significance. The application to cell painting and related assays is of broad enough significance for many journals, However, the authors could further broaden the significance by commenting on other possible cell biology applications. What other applications might the algorithm be particularly suited for? Are there any possible roadblocks to wider use. What sorts of data has the code been tested on so far?

We have added the following paragraph to discuss the broader applicability of CytoSummaryNet:

“The architecture of CytoSummaryNet holds significant potential for broader applications beyond image-based cell profiling, accommodating tabular, permutation-invariant data and enhancing downstream task performance when applied to processed population-level profiles. Its versatility makes it valuable for any omics measurements where downstream tasks depend on measuring similarity between profiles. Future research could also explore CytoSummaryNet's applicability to genetic perturbations, expanding its utility in functional genomics.”

Reviewer 3

R3.0 The authors have done a commendable job discussing the method, demonstrating its potential to outperform current models in profiling cell-based features. The work is of considerable significance and interest to a wide field of researchers working on the understanding of cell heterogeneity's impact on various biological phenomena and practical studies in pharmacology.

One aspect that would further enhance the value of this work is an exploration of the method's separation power across different modes of action. For instance, it would be interesting to ascertain if the method's performance varies when dealing with actions that primarily affect size, those that affect marker expression, or compounds that significantly diminish cell numbers.

Thank you for encouraging comments!

We have added the following to *Results: Relevance scores reveal CytoSummaryNet's preference for large, isolated cells*:

“Statistical t-tests were conducted to identify the features that most effectively differentiate mechanisms of action from negative controls in average profiles, focusing on the three mechanisms of action where CytoSummaryNet demonstrates the most significant improvement and the three mechanisms where it shows the least. Consistent with our hypothesis that CytoSummaryNet emphasizes larger, more sparse cells, the important features for the CytoSummaryNet-improved mechanisms of action (Supplementary Material I1) often involve the radial distribution for the mitochondria and RNA channels. These metrics capture the fraction of those stains near the edge of the cell versus concentric rings towards the nucleus, which are more readily detectable in larger cells compared to small, rounded cells.

In contrast, the important features for mechanisms of action not improved by CytoSummaryNet (Supplementary Material I) predominantly include correlation metrics between brightfield and various fluorescent channels, capturing spatial relationships between cellular components. Some of these mechanisms of action included compounds that were not individually distinguishable from negative controls, and CytoSummaryNet did not overcome the lack of phenotype in these cases. This suggests that while CytoSummaryNet excels in identifying certain cellular features, its effectiveness is limited when dealing with mechanisms of action that do not exhibit pronounced phenotypic changes.”

We have also added supplementary material to support (*I. Relevant features for CytoSummaryNet improvement*).

R3.0 Another test on datasets that are not concerned with chemical compounds, but rather genetic perturbations would greatly increase the reach of the method into the functional genomics community and beyond. This additional analysis could provide valuable insights into the versatility and applicability of the proposed method.

We agree that testing the method's behavior on genetic perturbations would be interesting and could provide insights into its versatility. However, the efficacy of the methodology may vary depending on the specific properties of different genetic perturbation types.

For example, the penetrance of phenotypes may differ between genetic and chemical perturbations. In some experimental setups, a selection agent ensures that nearly all cells receive a genetic perturbation (though not all may express a phenotype due to heterogeneity or varying levels of the target protein). Other experiments may omit such an agent. Additionally, different patterns might be observed in various classes of reagents, such as overexpression, CRISPR-Cas9 knockdown (CRISPRn), CRISPR-interference (CRISPRi), and CRISPR-activation (CRISPRa).

We believe that selecting a single experiment with one of these technologies would not adequately address the question of versatility. Instead, we propose future studies that may conclusively assess the method's performance across a variety of genetic perturbation types. This would provide a more comprehensive understanding of CytoSummaryNet's applicability in functional genomics and beyond. We have update the Discussion section to reflect this:

“Future research could also explore CytoSummaryNet's applicability to genetic perturbations, expanding its utility in functional genomics.”

R3.1. The datasets were stratified based on plates and compounds. It would be beneficial to clarify the basis for data stratification applied for compounds. Was the data sampled based on structural or functional similarity of compounds? If not, what can be expected from the model if trained and validated using structurally or functionally diverse and non-diverse compounds?

Thank you for raising the important question of data stratification based on compound similarity. In our study, the data stratification was performed by randomly sampling the compounds, without considering their structural or functional similarity.

This approach may limit the generalizability of the learned representations to new structural or functional classes not captured in the training set. Consequently, the current methodology may not fully characterize the model's performance across diverse compound structures.

In future work, it would be valuable to explore the impact of compound diversity on model performance by stratifying data based on structural or functional similarity and comparing the results to our current random stratification approach to more thoroughly characterize the learned representations.

R3.2. Is the method prioritizing a particular biological reaction of cells toward common chemical compounds, such as mitotic failure? Could this be oncology-specific, or is there more utility to it in other datasets?

Our analysis of CytoSummaryNet's performance in (the new) Figure 6 reveals a strong improvement in MoAs targeting cancer-related pathways, such as MEK, HSP, MDM, dehydrogenase, and purine antagonist inhibitors. These MoAs share a common focus on cellular proliferation, survival, and metabolic processes, which are key characteristics of cancer cells.

Given the composition of the cpg0004 dataset, which contains 1,258 unique MoAs with only 28 annotated as oncology-related, the likelihood of randomly selecting five oncology-related MoAs that show strong improvement is extremely low. This suggests that the observed prioritization is not due to chance.

Furthermore, the prioritization cannot be solely attributed to the frequency of oncology-related MoAs in the dataset. Other prevalent disease areas, such as neurology/psychiatry, infectious disease, and cardiology, do not exhibit similar improvements despite having higher MoA counts.

While these findings indicate a potential prioritization of oncology-related MoAs by CytoSummaryNet, further research is necessary to fully understand the extent and implications of this bias. Future work should involve conducting similar analyses across other disease areas and cell types to assess the method's broader utility and identify areas for refinement and application. However, given the speculative nature of these observations, we have chosen not to update the manuscript to discuss this potential bias at this time.

R3.3 Figures 1 and 2 demonstrate that the CytoSummaryNet profiles outperform average-aggregated profiles. However, the average profiling results seem more consistent when compared to CytoSummaryNet profiling. What further conditions or approaches can help improve CytoSummaryNet profiling results to be more consistent?

The observed variability in CytoSummaryNet's performance is primarily due to the intentional technical variance in our datasets, where each plate tested different staining protocol variations. It's important to note that this level of technical variance is not typical in standard cell profiling experiments. In practice, the variance across plates would be much lower. We want to emphasize that while a model capable of generalizing across diverse experimental conditions might seem ideal, it may not be as practically useful in real-world scenarios. This is because such non-uniform conditions are uncommon in typical cell profiling experiments. In normal experimental settings, where technical variance is more controlled, we expect CytoSummaryNet's performance to be more consistent.

R3.4 Can the poor performance on unseen data (in the case of stain 5) be overcome? If yes, how? If no, why not?

We believe that the poor performance on unseen data, such as Stain 5, can be overcome depending on the nature of the unseen data. As shown in Figure 4 (panel 3), the model improves upon average profiling for unseen data when the experimental conditions are similar to the training set.

The issue lies in the different experimental conditions. As explained in our response to R3.3, this could be addressed by including these experimental conditions in the training dataset. As long as CytoSummaryNet is trained (seen) and tested (unseen) on data generated under similar experimental conditions, we are confident that it will improve or perform as well as average profiling.

It's important to note that the issue of generalization to vastly different experimental conditions was considered out of scope for this paper. The main focus is to introduce a new method that improves upon average profiling and can be readily used within a consistent experimental setup.

R3.5 It needs to be mentioned how the feature data used for CytoSummaryNet profiling was normalized before training the model. What would be the impact of feature data normalization before model training? Would the model still outperform if the skewed feature data is normalized using square or log transformation before model training?

We have clarified in the manuscript that we standardized the feature data on a plate-by-plate basis to achieve zero mean and unit variance across all cells per feature within each plate. We have added the following statement to improve clarity:

“The data used to compute the average profiles and train the model were standardized at the plate-level, ensuring that all cell features across the plate had a zero mean and unit variance. The negative control wells were then removed from all plates."

We chose standardization over transformations like squaring or logging to maintain a balanced scale across features while preserving the biological and morphological information inherent in the data. While transformations can reduce skewness and are useful for data spanning several orders of magnitude, they might distort biological relevance by compressing or expanding data ranges in ways that could obscure important cellular variations.

Regarding the potential impact of square or log transformations on skewed feature data, these methods could improve the model's learning efficiency by making the feature distribution more symmetrical. However, the suitability and effectiveness of these techniques would depend on the specific data characteristics and the model architecture.

Although not explored in this study, investigating various normalization techniques could be a valuable direction for future research to assess their impact on the performance and adaptability of CytoSummaryNet across diverse datasets and experimental setups.

R3.6. In Figure 5 b and c, MoAs often seem to be represented by singular compounds and thus, the test (MoA prediction) is very similar to the training (compound ID). Given this context, a discussion about the extent this presents a circular argument supported by stats on the compound library used for training and testing would be beneficial.

Clusters in (the new) Figure 7 that contain only replicates of a single compound would not yield an improved performance on the MoA task unless they also include replicates of other compounds sharing the same MoA in close proximity. Please see our response to R1.1c.iii. for details. To improve visual clarity and avoid misinterpretation, we have recomputed the colors for (the new) Figure 7 and grayed out overlapping points.

R3.7 Can you estimate the minimum amount of supervision (fuzzy/sparse labels, often present in mislabeled compound libraries with dirty compounds and polypharmacology being present) that is needed for it to be efficiently trained?

It's important to note that the metadata used by the model is only based on identifying replicates of the same compound. Mechanism of action (MoA) annotations, which can be erroneous due to dirty compounds, polypharmacology, and incomplete information, are not used in training at all. MoA annotations are only used in our evaluation, specifically for calculating the mAP for MoA retrieval.

We have successfully trained CytoSummaryNet on 72 unique compounds with 4 replicates each. This is the current empirical minimum, but it is possible that the model could be trained effectively with even fewer compounds or replicates.

Determining the absolute minimum amount of supervision required for efficient training would require further experimentation and analysis. Factors such as data quality, feature dimensionality, and model complexity could influence the required level of supervision.

R3.minor1 Figure 5: The x-axis and y-axis tick values are too small, and image resolution/size needs to be increased.

We have made the following changes to address the concerns:

- Increased the image resolution and size to improve clarity and readability.
- Removed the x-axis and y-axis tick values, as they do not provide meaningful information in the context of UMAP visualizations.

We believe these modifications enhance the visual presentation of the data and make it easier for readers to interpret the results.

R3.minor2 The methods applied to optimize hyperparameters in supplementary data need to be included.

We added the following to Supplementary Material D:

"We used the Weights & Biases (WandB) sweep suite in combination with the BOHB (Bayesian Optimization and HyperBand) algorithm for hyperparameter sweeps. The BOHB algorithm [47] combines Bayesian optimization with bandit-based strategies to efficiently find optimal hyperparameters.

Additionally Table D1 provides an overview of all tunable hyperparameters and their chosen values based on a BOHB hyperparameter optimization."

R3.minor3 Figure 5(c, d): The names of compound 2 and Compound 5 need to be included in the labels.

These compounds were obtained from external companies and are proprietary, necessitating their anonymization in our study. This has now been added in the caption of (the new) Figure 7:

"Note that Compound2 and Compound5 are intentionally anonymized."

R3.minor4 Table C1: Plate descriptions need to be included.

Table C1: The training, validation, and test set stratification for Stain2, Stain3, Stain4, and Stain5. Five training, four validation, and three test plates are used for Stain2, Stain3, and Stain4. Stain5 contains six test set plates only.

Stain2	Stain3	Stain4	Stain5
Training plates			*Test plates*
BR00113818	BR00115128	BR00116627	BR00120532
BR00113820	BR00115125highexp	BR00116631	BR00120270
BR00112202	BR00115133highexp	BR00116625	BR00120536
BR00112197binned	BR00115131	BR00116630highexp	BR00120530
BR00112198	BR00115134	200922_015124-Vhighexp	BR00120526
Validation plates			BR00120274
BR00112197standard	BR00115129	BR00116628highexp	
BR00112197repeat	BR00115133	BR00116629highexp	
BR00112204	BR00115128highexp	BR00116627highexp	
BR00112201	BR00115127	BR00116629	
Test plates			
BR00112199	BR00115134bin1	200922_044247-Vbin1	
BR00113819	BR00115134multiplane	200922_015124-V	
BR00113821	BR00115126highexp	BR00116633bin1	

We have added a reference to the metadata file in the description of Table C1: https://github.com/carpenter-singh-lab/2023_Cimini_NatureProtocols/blob/main/JUMPExperimentMasterTable.csv

R3.minor5 Figure F1: Does the green box (stain 3) also involve training on plates from stain 4 (BR00116630highexp) and 5 (BR00120530) mentioned in Table C1? Please check the figure once again for possible errors.

We have carefully re-examined Figure F1 and Table C1 to ensure their accuracy and consistency. Upon double-checking, we can confirm that the figure is indeed correct. We intentionally omitted the training and validation plates from Figure F1 to maintain clarity and readability, as including them resulted in a cluttered and difficult-to-interpret figure.

Regarding the specific plates mentioned:

- BR00116630highexp (Stain4) is used for training, as correctly stated in Table C1. This plate is considered an outlier within the Stain4 dataset and happens to cluster with the Stain3 plates in Figure F1.
- BR00120530 (Stain5) is part of the test set only and correctly falls within the Stain5 cluster in Figure F1.

To improve the clarity of the training, validation, and test split in Table C1, we have added a color scheme that visually distinguishes the different data subsets. This should make it easier for readers to understand the distribution of plates across the various splits.
